# Supplementary material for: Differences in Disability Perception in Systemic Sclerosis: A Mirror Survey of Patients and Health Care Providers
Source: J Clin Med. 2023 Feb 8;12(4):1359. doi: 10.3390/jcm12041359 (PMC9959687; doi:10.3390/jcm12041359)
Supplement: Supplementary file 1 [file jcm-12-01359-s001.zip › jcm-2172258-supplementary.pdf]

Table S1: Checklist for Reporting Results of Internet E-surveys (CHERRIES).

Item Category	Checklist Item	Explanation
Design	Describe survey design	It was a closed-survey. The target population and sample frame were French patients of the online SPIN Cohort
Institutional Review Board (IRB) approval and informed consent process	IRB approval	Approved by our IRB (*Comité de Protection des Personnes Île-de-France I*)
	Informed consent	Patients from the SPIN Cohort gave their written informed consent to participate in the Cohort. Along with the link to the online questionnaires, patients received an electronic note to inform them about the specific research question addressed in the present study
	Data protection	For the provisional questionnaire, no personal information was collected in addition to the questionnaires answers, and answers were stored on a secured server at the clinical research unit.<br>For the test-retest, name, gender and answer to the questionnaire were collected and stored on a secured Google drive.
Development and pre-testing	Development and testing	Online version of the provisional questionnaire was developed and its functionality was tested in July 2017.<br>The online version of the final questionnaire for the test-retest was developed and tested in May 2018.
Recruitment process and description of the sample having access to the questionnaire	Open Survey vs closed survey	Closed survey, a secured link and personalized password were send to each participant.
	Contact mode	Initial contact was made by mail.
	Advertising the survey	No advertising was made.
Survey administration	Web/E-mail	The questionnaires were stored on a website, with automatic method for capturing responses in the database.
	Context	The survey was on the website of the clinical research unit of Paris Descartes:<br>http://www.rechercheclinqueparíscentre.fr.<br>Only participants contacted by mail received the link to the secured online platform.
	Mandatory/voluntary	It was a voluntary survey.
	Incentives	No incentives were used.
	Time/Date	For the provisional questionnaire : from February 6, 2018 to March 31, 2018<br>For the test-retest : from May 16, 2018 to June 20, 2018
	Randomization of items or questionnaires	No randomization planned or needed for the purpose of this study.
	Adaptative questioning	No randomization planned or needed for the purpose of this study.
	Number of items	For the provisional questionnaire: 155 items, distributed as follows:<br>- Provisional scale: 65 items<br>- HAQ : 20 items<br>- sHAQ: 5 items<br>- MACTAR: 7 items

		- CHFS: 18 items<br>- MHISS: 12 items<br>- HADS: 14 items<br>- SF-12: 12 items<br>- NRS pain: 1 item<br>- NRS aesthetic burden: 1 item<br>For the test-retest : 17 items
	Number of screens (pages)	For the provisional questionnaire : 26 pages<br>For the test-retest : 1 page
	Completeness check	For the provisional questionnaire, completeness was checked after the questionnaire has been submitted. No item was mandatory.<br>For the test-retest, completeness was checked before the questionnaire has been submitted, all the items were mandatory.
	Review step	Patients were able to change their answers with a review step, and they had the possibility to have several accesses to complete or modify their answers.
Response rates	Unique site visitor	Each participant had a unique identifying code and a personalized access-link and password. Patients' answers were saved under their identifying codes.
	View rate (ratio unique site visitors/unique survey visitors)	N/A; only patients of the survey could access to the internet platform.
	Participation rate (ratio unique survey page visitors/agreed to participate)	For the provisional questionnaires: 113/184 invited participants answered (61.4% of answer rate)<br>For the test-retest: 34/75 (45.3%) and 24/34 (70.6%) invited participants answered
	Completion rate (ratio agreed to participate/finished survey)	For the provisional questionnaire: 109/113 (96.4%) completed the provisional questionnaire (subject to item ratio of 1.7) and 85/113 (75.2%) at least another questionnaire in addition to the provisional questionnaire.<br>For the test-retest, the completion rate was 100% (34/34 for the test and 24/24 for the retest).
Preventing multiple entries from the same individual	Cookies used	No cookies were used.
	IP check	IP addresses were not checked.
	Log file analysis	N/A
	Registration	Patient's answers were registered under his identifying code, given by the personalized access-link and password. Multiples entries were allowed, patients could change their answers and the last answer was kept for analysis.
Analysis	Handling of incomplete questionnaires	For the provisional scale an item without answer was assumed irrelevant. For the other scales, in case of a missing item, the questionnaire was not examined, excepted for the SF-12 and MACTAR where imputations performed.<br>No incomplete questionnaire was allowed for the test-retest.
	Questionnaires submitted with atypical	The time needed to fill in a questionnaire was not used to exclude questionnaires.

timestamp	
Statistical correction	N/A

Table S2. Cochin Scleroderma ICF-65 questionnaire for activity and participation in patients with systemic sclerosis.

**Learning and applying knowledge**
Because of my systemic sclerosis*, *I feel limited in the following daily activities:
Q1. Learning new things?
Q2. Focusing my attention?
Q3. Reading?
Q4. Writing with a pen or a pencil?
**General tasks and demands**
Because of my systemic sclerosis*, *I feel limited in the following daily activities:
Q5. Solving problems of daily life?
Q6. Making decisions?
Q7. Undertaking a complex task requiring several steps?
Q8. Undertaking multiple simultaneous or successive tasks?
Q9. Carrying out daily routine (planning, carrying out, undertaking tasks and demands of daily life)?
Q10. Managing my own activity level?
Q11. Handling stress and other psychological demands?
Q12. Handling responsibilities in my personal and professional life?
**Mobility**
Because of my systemic sclerosis*, *I feel limited in the following daily activities:
Q13. Changing my body position?
Q14. Kneeling down, squatting alone?
Q15. Standing up alone?
Q16. Bending forward?
Q17. Maintaining a lying position?
Q18. Maintaining a standing position?
Q19. Lifting and carrying objects in my hands even when moving?
Q20. Manipulating small objects using my fingers and hands?
Q21. Moving arms (raise, flex, extend)?
Q22. Pulling or pushing an object?
Q23. Walking?
Q24. Moving downwards (a step, a slope, a ladder)?
Q25. Moving upwards or downwards (a step, a stool, a slope, a ladder)?

Q26. Running?

Q27. Going somewhere (inside and outside the home)?

Q28. Taking a plane or a train?

Q29. Using public transportation (bus, metro, tramway)?

Q30. Driving (a car, a motocycle, a bicycle)?

Self-care

***Because of my systemic sclerosis*, I feel limited in the following daily activities:**

Q31. Washing myself?

Q32. Caring for my physical appearance (combing, shaving, removing hair, brushing teeth, caring for skin, hands, feet, making up, choosing my clothes)?

Q33. Toileting?

Q34. Putting on clothes, taking off clothes, putting on footwear, taking off footwear?

Q35. Eating?

Q36. Looking after my health?

Domestic life

***Because of my systemic sclerosis*, I feel limited in the following daily activities:**

Q37. Shopping?

Q38. Preparing meals?

Q39. Doing housework (washing dishes, washing clothes, housekeeping, ironing, cleaning)?

Q40. Thinkering, gardening, feeding and taking care of my domestic animals?

Q41. Assisting others (familly members, neighbours, relatives) according to their needs?

Communication

***Because of my systemic sclerosis*, I feel limited in the following daily activities:**

Q42. Expressing myself and making myself understood in oral language?

Q43. Starting a conversation or conversing with one person or many people?

Q44. Using a landline or a mobile phone?

Q45. Using a computer (reading the computer screen and/or writing using a keyboard)?

Interpersonal interactions and relationships

***Because of my systemic sclerosis*, I feel limited in the following daily activities:**

Q46. Interacting with someone in a contextually and socially appropriate manner?

Q47. Accepting bodily contact (allowing physical contact, hugging)?

Q48. Forming and terminating relationships?

Q49. Regulating emotions, verbal aggression and physical aggression in interactions with others?

Q50. Engaging in contacts with strangers for specific purposes (asking for directions, making a purchase, reporting a problem)?

Q51. Having and maintaining relationships with friends?

Q52. Having and maintaining relationships with the members of my family?

Q53. Creating and maintaining close or romantic relationships with someone?

Q54. Having a satisfying sexual life?

Major life areas

***Because of my systemic sclerosis*, I feel restricted in participating in the following daily activities:**

Q55. Taking an exam?

Q56. Engaging in an educational program (being present, being diligent)?

Q57. To seek, to change, to find or to keep a job?

Q58. Doing all the required tasks and activities of my job?

Q59. Working full-time?

Community, social and civic life

***Because of my systemic sclerosis*, I feel restricted in participating in the following daily activities:**

Q60. Travelling in France or overseas?

Q61. To do sport?

Q62. Going to cultural events (shows, museums, exhibitions)?

Q63. Doing handicrafts (sewing, collections, craftwork)?

Q64. Having and developing my spiritual life?

Q65. Participating in local and political life as a citizen (vote, local debate, unionism)?

NOTE. The items were originally in the French language.
